# Whole Genome Sequencing Refines Knowledge on the Population Structure of *Mycobacterium bovis* from a Multi-Host Tuberculosis System

**DOI:** 10.3390/microorganisms9081585

**Published:** 2021-07-26

**Authors:** Ana C. Reis, Liliana C. M. Salvador, Suelee Robbe-Austerman, Rogério Tenreiro, Ana Botelho, Teresa Albuquerque, Mónica V. Cunha

**Affiliations:** 1 Centre for Ecology, Evolution and Environmental Changes (cE3c), Faculdade de Ciências da Universidade de Lisboa, 1749-016 Lisboa, Portugal; ana.reis714@gmail.com; 2 Biosystems & Integrative Sciences Institute (BioISI), Faculdade de Ciências da Universidade de Lisboa, 1749-016 Lisboa, Portugal; rptenreiro@fc.ul.pt; 3 Department of Infectious Diseases, College of Veterinary Medicine, University of Georgia, Athens, GA 30602, USA; salvador@uga.edu; 4 Institute of Bioinformatics, University of Georgia, Athens, GA 30602, USA; 5 Center for the Ecology of Infectious Diseases, University of Georgia, Athens, GA 30602, USA; 6 USDA/APHIS National Veterinary Services Laboratories, Ames, IA 50010, USA; suelee.robbe-austerman@usda.gov; 7 INIAV, IP-National Institute for Agrarian and Veterinary Research, 2780-157 Oeiras, Portugal; ana.botelho@iniav.pt (A.B.); teresa.albuquerque@iniav.pt (T.A.)

**Keywords:** animal tuberculosis, multi-host transmission, *Mycobacterium bovis*, whole-genome sequencing, wildlife-livestock interface

## Abstract

Classical molecular analyses of *Mycobacterium bovis* based on spoligotyping and Variable Number Tandem Repeat (MIRU-VNTR) brought the first insights into the epidemiology of animal tuberculosis (TB) in Portugal, showing high genotypic diversity of circulating strains that mostly cluster within the European 2 clonal complex. Previous surveillance provided valuable information on the prevalence and spatial occurrence of TB and highlighted prevalent genotypes in areas where livestock and wild ungulates are sympatric. However, links at the wildlife–livestock interfaces were established mainly via classical genotype associations. Here, we apply whole genome sequencing (WGS) to cattle, red deer and wild boar isolates to reconstruct the *M. bovis* population structure in a multi-host, multi-region disease system and to explore links at a fine genomic scale between *M. bovis* from wildlife hosts and cattle. Whole genome sequences of 44 representative *M. bovis* isolates, obtained between 2003 and 2015 from three TB hotspots, were compared through single nucleotide polymorphism (SNP) variant calling analyses. Consistent with previous results combining classical genotyping with Bayesian population admixture modelling, SNP-based phylogenies support the branching of this *M. bovis* population into five genetic clades, three with apparent geographic specificities, as well as the establishment of an SNP catalogue specific to each clade, which may be explored in the future as phylogenetic markers. The core genome alignment of SNPs was integrated within a spatiotemporal metadata framework to further structure this *M. bovis* population by host species and TB hotspots, providing a baseline for network analyses in different epidemiological and disease control contexts. WGS of *M. bovis* isolates from Portugal is reported for the first time in this pilot study, refining the spatiotemporal context of TB at the wildlife–livestock interface and providing further support to the key role of red deer and wild boar on disease maintenance. The SNP diversity observed within this dataset supports the natural circulation of *M. bovis* for a long time period, as well as multiple introduction events of the pathogen in this Iberian multi-host system.

## 1. Introduction

*Mycobacterium bovis* is an important pathogen, responsible for causing animal tuberculosis (TB) in livestock and wildlife vertebrates, as well as in humans [1,2]. Cattle (*Bos taurus*) is the main livestock affected species, while several reports evidence the importance of the livestock–wildlife interface for disease maintenance [3,4,5,6]. In the Iberian Peninsula, red deer (*Cervus elaphus*) and wild boar (*Sus scrofa*) have both been implicated in the transmission of *M. bovis* to cattle via direct and indirect routes and in pathogen persistence across ecosystems, depending on the specificities of the epidemiological scenario and the ecological relationships established by the hosts [7,8,9,10,11,12]. The presence of maintenance hosts in the wild is associated with difficulties in the success of test and slaughter schemes implemented in the cattle population, but it also brings concerns regarding wildlife welfare, biodiversity and public health. Most eradication programs in place are focused on cattle and based on test-and-slaughter approaches, movement restrictions and post-mortem surveillance at slaughterhouses [13]. Currently, an eradication program for animal TB in the cattle population is implemented in mainland Portugal and in the autonomous regions of Azores and Madeira. The program is based on the detection and slaughter of reactors to the single intradermal comparative cervical tuberculin test (SICCT), routine surveillance at slaughterhouses, mandatory sanitary classification of herds and regions, compulsory slaughter of reactors, monetary compensation to owners of slaughtered animals and pre-movement testing [14]. The Algarve, in the extreme south of Portugal, is the single region in Portugal that has already achieved the officially TB-free status (Decision 2012/204/EU), while in the remaining territory the epidemiological indicators have remained low in the past years, with TB prevalence registering 0.29% and animal prevalence 0.045% in 2017, according to the latest public report [15]. When considering wildlife species, passive surveillance measures have been applied since 2011 on hunted-harvested red deer and wild boar in a defined epidemiological risk area for big game species located in south-central Portugal [16]. All big game specimens hunted in this risk area are mandatorily subjected to post-mortem examination in the field by a credentialed veterinarian. Therefore, in Portugal, the trends in epidemiological indicators have led to adaptive surveillance and control measures laid upon different host species and regions [12,13]. To date, works with reference to *M. bovis* molecular characterization in Portugal have been based on the analysis of repetitive genomic regions, namely spoligotyping (spacer oligonucleotide typing) and MIRU-VNTR (*Mycobacterial Interspersed Repetitive Units-Variable Number Tandem Repeats*). The focus has been placed over isolates from cattle, red deer and wild boar from TB hotspot areas located in the central and southern regions of the country [12,17,18]. These works provided evidence for *M. bovis* population diversity and structure, highlighting the main genotypes across host species and regions, as well as intra- and inter-specific transmission [12,17,18]. However, these molecular typing approaches have explored epidemiological links via genotype associations, which are not sufficiently discriminatory to accurately assess transmission at a fine-scale, nor to gain insights on the roles exerted by different species in a multi-host system. However, understanding the evolutionary processes driving transmission among sympatric wildlife reservoirs and livestock populations is crucial for the effective management of animal TB in an endemic system.

The progressive application of whole genome sequencing (WGS) to infectious disease systems has resulted in unprecedented advances in the ability to resolve epidemiological information at different scales. WGS data provides higher discriminatory power than classical molecular approaches for resolving complex outbreak situations, allowing a finer definition of the spatiotemporal context in which pathogen spread and persistence occurs. WGS also aids in the identification of the infection source, the establishment of epidemiological links, and the reconstruction of transmission chains [19,20,21].

When considering the livestock–wildlife interface, WGS has been used to demonstrate the close genetic relationship among *M. bovis* isolates recovered from sympatric cattle and wildlife populations in different epidemiological settings, including the UK [19,22,23], Ireland [24], New Zealand [21,25] and the United States of America [20,26]. In this context, single nucleotide polymorphisms (SNPs) emerged as good phylogenetic markers, helping in the definition of *M. bovis* population structure, having been recently used to define four *M. bovis* lineages, and to inform transmission models [19,21,27]. When placed together with data concerning the time needed for this slow-growing bacterium to accumulate new SNPs, this information can provide temporal clues on the emergence and divergence of specific genotypes.

With the aim to improve knowledge on the *M. bovis* population structure within and across TB hotspots in Portugal, a pilot study involving WGS of 44 *M. bovis* obtained between 2003 and 2015 from cattle, red deer and wild boar was completed in this work. These isolates were selected as being representative of the *M. bovis* population diversity in those areas, which was previously assessed by a large-scale genotyping study that combined standard genotyping techniques (spoligotyping and MIRU-VNTR) with Bayesian clustering [18]. The methodological framework aimed to: (1) identify phylogenetic clades and build a catalogue of SNPs that may be used as specific molecular markers of each clade; (2) explore how specific nucleotide differences are associated with distinct host and/or geographic regions; and (3) perform phylogeographic analyses built upon networks linking the main hosts and TB hotspots in Portugal during different time frames of the animal TB control plan in Portugal.

## 2. Materials and Methods

### 2.1. M. bovis Isolates Dataset

The 44 *M. bovis* isolates used in this work were recovered from cattle (*n* = 16), red deer (*n* = 16) and wild boar (*n* = 12) from three TB hotspot areas in Portugal that encompass the districts (administrative level sample unit) of Castelo Branco, Portalegre and Beja, which are located in inner central and south of the mainland territory (Supplementary Figure S1 and Supplementary Table S1). This dataset was selected for WGS from a wider *M. bovis* dataset recovered in Portugal (*n* = 487) that was previously submitted for molecular characterization by classical genotyping techniques, namely spoligotyping and 8 *loci* MIRU-VNTR (Supplementary Figure S1) [18]. To represent the population genetic diversity, two selection criteria were applied: first, isolates should represent major spoligotyping-MIRU type groups and adjacent variants; this selection was performed with the help of Minimum Spanning Trees obtained for each geographic region using Bionumerics software (see below); and second, they should cover different host species, geographic regions and temporal/epidemiological contexts (using the year of *M. bovis* isolation as proxy) (Supplementary Figure S1).

### 2.2. Ethical Approval

The *M. bovis* dataset analyzed here was selected for WGS from a wider *M. bovis* dataset recovered in Portugal [18] in the scope of official control plans for animal TB. No animals were sacrificed for the purposes of this study. Isolates were obtained from the national reference laboratory of animal tuberculosis (INIAV, IP), from animal samples either presenting TB-compatible lesions during official inspection and/or animal samples from reactor cattle submitted for an official standard screening test for TB (the single intradermal comparative cervical tuberculin (SICCT) test). None of the authors were responsible for the death of any animals nor were any samples used in the study collected by the authors. All applicable institutional and/or national/international guidelines for the care and use of animals have been followed.

### 2.3. DNA Extraction

Bacteriological culture was performed as described by Reis et al. (2020). Frozen culture stocks of 44 *M. bovis* were successfully re-cultured on Middlebrook 7H9 (Difco) medium supplemented with 5% sodium pyruvate and 10% ADS enrichment (50 g albumin, 20 g glucose, 8.5 g sodium chloride in 1 L water) at 37 °C in a level 3 biosecurity facility.

After four weeks’ growth, the culture medium was renewed, and the cultures were monitored regularly until growth was observed. Cells were harvested, centrifuged and the culture pellet was re-suspended in 500 µL PBS and inactivated by heating at 99 °C for 30 min. After centrifugation, the supernatants were stored at −20 °C until further use.

### 2.4. Whole-Genome Sequencing and SNP Analysis

The genomic DNA was sequenced using the Illumina Genome Analyser, according to the manufacturer’s specifications, with the paired-end module attachment. Forty-two samples were sequenced by MiSeq technology (2 × 250 pb) at the United States Department of Agriculture (USDA, Ames, IA, USA) and the remaining two by HiSeq (2 × 150 pb) (Eurofins, Konstanz, Germany).

The vSNP pipeline, currently available at https://github.com/USDA-VS/vSNP, was used to process the FASTQ files obtained from Illumina sequencing (accessed on 1 September 2019). Briefly, reads were aligned to the *M. bovis* AF2122/97 reference genome (NCBI accession number LT708304.1) using BWA and Samtools [28,29]. Base quality score recalibration, SNP and indel (insertion or deletion) discovery were applied across all isolates using standard filtering parameters or variant quality score recalibration according to Genome Analysis Toolkit (GATK)’s Best Practices recommendations [30,31,32]. Results were filtered using a minimum SAMtools quality score of 150 and AC = 2.

The Integrated Genomics Viewer (IGV) (version 2.4.19) [33] was used to visually validate SNPs and positions with mapping issues or alignment problems. SNPs that fell within Proline-Glutamate (PE) and Proline-Proline Glutamate (PPE) genes were filtered from the analysis, as well as indels.

The raw data are deposited in a public domain server at the National Centre for Biotechnology Information (NCBI) SRA database, under BioProject accession number PRJNA682618.

### 2.5. Phylogenetic Analysis

Validated and polymorphic SNPs were concatenated, resulting in a single 1842-nt sequence. MEGA (Molecular Evolutionary Genetics Analysis, version 7.0) [34] was used to conduct phylogenetic analysis, using the maximum likelihood method with 1000 bootstrap inferences.

The distribution of pairwise SNP distance was obtained by applying the Hamming distance, using the library ape, and the corresponding heatmap was obtained by library gplots, both in the R statistical package [35].

The BioNumerics software (version 6.6, https://www.applied-maths.com/bionumerics, accessed on 1 September 2019) (Applied Maths, Saint-Martens-Latem, Belgium) was used to construct the dendrogram, using a combined dataset composed by spoligotyping and MIRU-VNTR profiles of the 44 *M. bovis*. A composite character experiment was created with spoligotyping and MIRU-VNTR data and an advanced cluster analysis was performed, using the categorical option, with the profiles of both techniques assuming the same weight. The Unweighted Pair Group Method with Arithmetic Mean (UPGMA) algorithm was selected for clustering.

### 2.6. Phylogeographic Analyses

A phylogeographic analysis was performed with a combined approach including Gephi version 0.9.2 [36] and QGIS (Quantum GIS development Team 2018, version 3.10).

A network to explore the relationships established between *M. bovis* isolates using the SNP pairwise distance as proxy was visualized in Gephi for three cumulative time periods and plotted in a map with QGIS. Each *M. bovis* was set as a node and the number of shared SNPs as connection lines, using Gephi default import criteria. Geographic coordinates were associated to each node. Three networks were generated with cumulative temporal intervals, resulting in the definition of period 1 (2003–2009), period 2 (2003–2012) and period 3 (2003–2015). These temporal windows are important from an epidemiological standpoint as they follow the variation of animal TB epidemiological indicators in Portugal, considering herd prevalence values in cattle, and the surveillance measures progressively implemented in wildlife (Supplementary Figure S2); in period 1, the values of herd prevalence steadily decreased, in period 2, an increase in herd prevalence was registered and carcass examination of hunted big game species became mandatory in the epidemiological risk area that is under analysis in this study, and, finally, in the last years added to the timeline, a decrease in herd prevalence was observed (Supplementary Figure S2) [12,13]. The connection lines were established based on absolute values of shared SNPs; no statistical transformation was performed.

A total of 36 *M. bovis* had information concerning geographical location: each *M. bovis* isolate from cattle was assigned to the geographic coordinates of the corresponding livestock herd; isolates from hunter-harvested red deer and wild boar were associated to the centroids of officially-delimited hunting areas. For eight *M. bovis* strains (cattle (*n* = 5), wild boar (*n* = 2), red deer (*n* = 1)), the specific geographic coordinates were absent, so the coordinates were assumed as the centroid of the geographic region so that all *M. bovis* could be plotted in the map. No substantial change in geographic coordinates was assumed over the study period.

### 2.7. Isolation by Distance Dissimilarity Analysis

A Mantel test was conducted in the R software environment package ade4 [37] to assess the relationship between spatial distances and SNP distances, using 10,000 permutations to assess significance. Only the isolates with geographical coordinates were included in this analysis (*n* = 36). To apply the Mantel test, a transformation of the distance matrices with geographic coordinates and the SNP alignment was applied in order to obtain dissimilarity matrices for both variables. Dissimilarity was expressed as the proportion of the maximum geographic distance or maximum number of SNPs, respectively, varying from 0 to 1.

## 3. Results

### SNP-Based Genotyping and Phylogenetic Analyses

The sequence reads of 44 *M. bovis* whole genomes representing the genetic diversity of strains circulating in TB hotspots in Portugal were mapped to the assembled reference genome of *M. bovis* AF2122/97 (LT708304.1) (Supplementary Table S1). The average depth of coverage and genome coverage were 93.6 and 99.57%, respectively (Supplementary Table S2). The SNP alignment had a total of 1842 polymorphic positions, with the majority (86.5%) located in coding regions.

**Figure 1:**
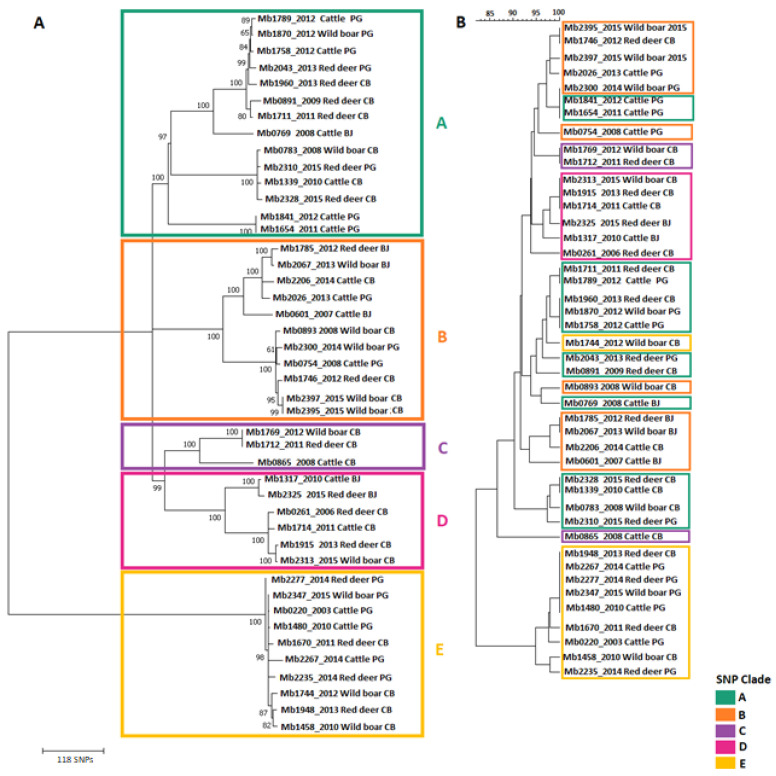
Genetic relatedness of *M. bovis* field strains from TB hotspots in Portugal. (**A**) Maximum Likelihood Tree using GTR model with input taken as an alignment file containing only informative and validated SNPs. The tree is drawn to SNP scale. The bootstrap values are represented. (**B**) UPGMA tree, applying categorical option as a similarity coefficient, with input taken as the combined dataset based on spoligotyping and 8-*loci* MIRU-VNTR data. Colors identify the different SNP clades (A—dark cyan, B—orange, C—purple, D—pink and E—yellow).

The phylogenetic distribution of SNPs grouped *M. bovis* into five related clades, each one with more than 100 clade-defining SNP sites, i.e., polymorphic positions specifically found within each clade member (Figure 1A and Table 1). Clades were named from A to E, being clade A the largest, counting with 14 *M. bovis* genomes, while clade C is the smallest, encompassing only three (Figure 1A).

The topology of the SNP-based phylogenetic tree differs from the topology of the tree built upon the combination of classical genotyping techniques outputs (spoligotyping patterns and MIRU-VNTR allelic profiles); however, the large branch division between clades A–D and clade E is maintained in both representations, suggesting that clade E has evolved independently from the remainder. While in the phylogenetic representation (Figure 1A), clades A and B are well structured, members from these clades are scattered in the dendrogram (Figure 1B) among several branches. In contrast, the clustering of members from clades D or E is well preserved among both trees (Figure 1A,B).

The majority of *M. bovis* isolates (*n* = 34, 77%, clades A to D) cluster within the highly structured clonal complex European 2 (Eu2) [38] that is widely distributed in the Iberian Peninsula. All members of these clades (A to D) possessed the *guaA* gene (G→A) synonymous mutation, a hallmark of Eu2 [38]. The remaining isolates were not assigned to any of the described clonal complexes. In agreement, Zimpel and collaborators noted multiple isolates from their global survey that did not fit into the previously established clonal complex categories and according to their SNP-based phylogeny with over 1900 *M. bovis*, they proposed a new four lineage taxonomy scheme for *M. bovis* [39]. Hauer and collaborators performed whole genome sequencing of *M. bovis* isolates from pre-established clonal complexes and covering the genetic diversity of French strains. They provided a global phylogeny picture based on SNP analyses and identified phylogenetic markers that greatly improved group resolution in comparison to the combination of spoligotyping and MLVA [40].

The clades in the upper phylogenetic branch (clades A to D) registered between 108 to 217 clade-defining SNP sites, while the lower phylogenetic branch (clade E) presented a total of 360 SNPs (Table 1). Moreover, for clades C, D and E, it was also possible to identify clade-monomorphic SNP sites (i.e., polymorphic positions present only in clade members and common to them all): 49 SNPs in clade C, 82 in clade D and 352 in clade E (Table 1). When accounting for the total SNP sites registered per clade, intra-clade homogeneity (i.e., the proportion of monomorphic SNP sites within each clade) ranged from 0% to 82%, in decreasing order: clade E (82%), clade D (22%), clade C (15%) and clades A and B (0%), pointing out clade E as the most homogeneous. If considering geographic region or host species as grouping criteria, group-defining SNP sites could also be identified for all categories under analysis; however, no monomorphic SNP sites were identified per host species or geographic region.

**Figure 2:**
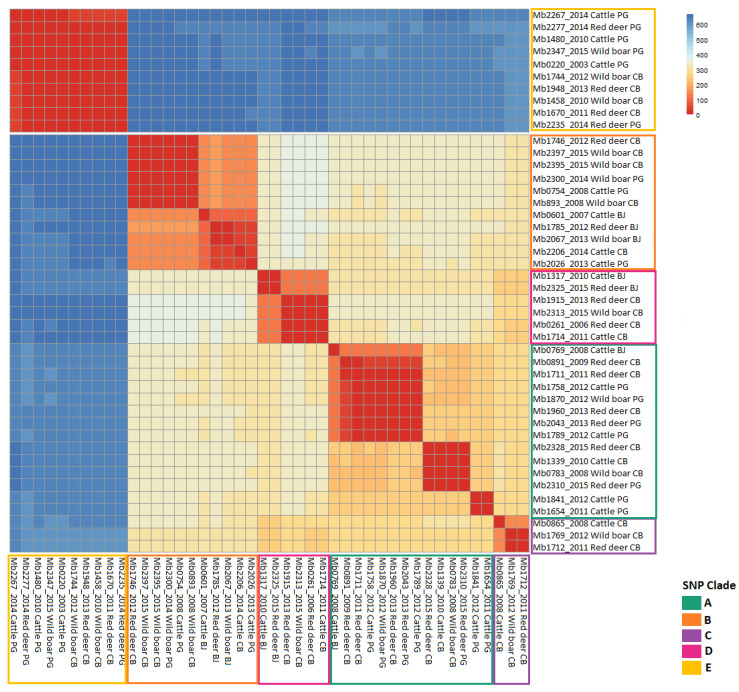
Heatmap of pairwise SNP distances based on the absolute distance of SNPs. The color of the boxes identifies the different SNP clades (A—dark cyan, B—orange, C—purple, D—pink and E—yellow).

The differences between phylogenetic branches are also clearly expressed in a heatmap based on the absolute SNP distance between strains, which supports a very clear separation between the members of clades A to D and clade E (Figure 2). Grouping by host species revealed that the mean genetic distance within each group is similar (382 SNPs within cattle; 397 SNPs within red deer; and 398 within wild boar), while dissimilarities strike out when comparing the three geographic regions covered by the analyses (376 SNPs within Castelo Branco; 405 within Portalegre; and 244 within Beja) (data not shown).

**Figure 3:**
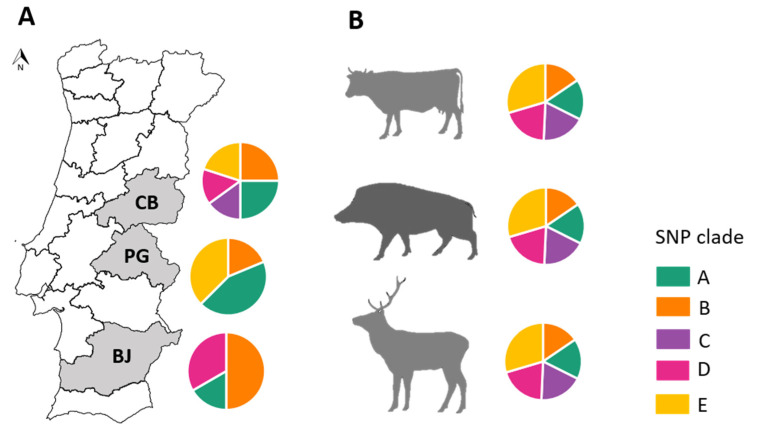
Distribution of SNP clades by: (**A**) geographic region (Beja (BJ), Castelo Branco (CB) and Portalegre (PG)) and (**B**) host species. Pie charts represent the proportion of each SNP clade by geographic region or host species. The colors of the pie charts identify the different SNP clades (A—dark cyan, B—orange, C—purple, D—pink and E—yellow).

Clades A and B were the most widely distributed, being found across the three TB hotspots under study. Clade C was exclusive to Castelo Branco, clade D was found to be absent in Portalegre and clade E was absent in Beja (Figure 3). In contrast, similar clade distributions were found at the host species level, with strains from the three hosts clustering in the five clades (Figure 3).

**Figure 4:**
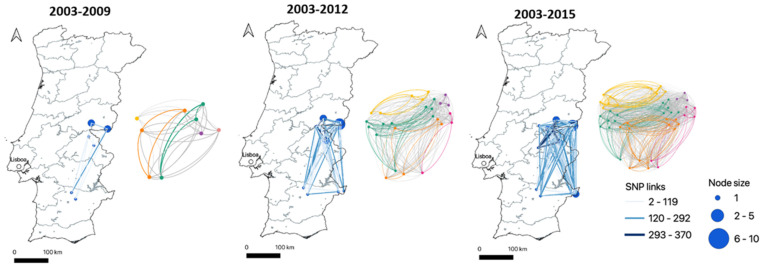
Temporal evolution of the SNP network established for the 44 *M. bovis* recovered between 2003 and 2015. Three cumulative time periods were considered according to the epidemiological scenarios in Portugal (see methods). In the map of Portugal, the nodes represent *M. bovis* strains and the complexity of connections is based on the number of shared SNPs between strains. The side network evidences the relationship established between *M. bovis* grouped within the same clade—each node represents one *M. bovis* strain and the colors identify the connections according to SNP clade (A—dark cyan, B—orange, C—purple, D—pink, E—yellow and grey for connections between different clades).

The temporal evolution of the established SNP network was assessed for multiple time periods to obtain insights into the strength of relationships established through space and time by *M. bovis* strains; therefore, the number of shared SNPs was used as a connection link (Figure 4). This approach includes three consecutive temporal windows according to the epidemiological phases of the animal TB enzootics in Portugal (Figure 4, Supplementary Figure S2). Based on this SNP dataset, each clade could not be distinguished from the others based solely on the sampling time of the isolates (Supplementary Figure S3). Furthermore, this analysis highlighted strong global and local networks in Portugal, with a particular focus on the strength of connections established within geographic regions (maximum shared SNPs between strains in Beja = 265, Castelo Branco = 370 and Portalegre = 365) and between Castelo Branco and Portalegre (*n* = 365) (Figure 4).

Links with zero SNP differences were identified within the same host species (cattle–cattle and wild boar–wild boar) and between different host species (red deer–wild boar), suggesting intra- and inter-specific transmission events.

A comparison of pairwise genetic distance (SNPs) and geographic distance through dissimilarity matrices was performed, only including cases with accurate geographic coordinates (*n* = 36). The Mantel test revealed that the geographic distance is not related to the genetic distance (simulated *p*-value = 0.541).

## 4. Discussion

This report describes the results from a pilot study applying for the first time WGS approaches to a restricted, but diverse, collection of 44 *M. bovis* from Portugal. The aims were to explore the fine-scale genomic signatures of field isolates, to highlight the value of phylogenetic inference and phylogeographic network approaches to understand the local history of this pathogen at the livestock–wildlife interface and to provide the foundations for future works with larger *M. bovis* datasets.

In the current research, the SNP-based phylogeny established five main clades, with clades C, D and E presenting lower levels of intra-clade diversity and clade-specific monomorphic SNP sites that can be explored as phylogenetic markers in future diagnostic and epidemiological studies. The topology of the SNP-based phylogenetic tree does not fully agree with the dendrogram obtained by combining classical genotyping methods, being, however, evident that the partition between clades A–D and clade E remains well-structured and that the members of clades D and E remain together. Other works have registered varying clustering agreement between spoligotyping and WGS [40,41], reinforcing the need to refine molecular epidemiology studies using next-level genomic scale methods. The combined analysis of spoligotyping and MIRU-VNTR profiles in a specific epidemiological scenario is useful for targeting regions for WGS analysis that might be undergoing a micro-epidemic of potentially linked cases.

The accuracy of the dendrogram based on combined spoligotyping and MIRU-VNTR patterns may be compromised by homoplasy (i.e., shared characters that did not arise from a common ancestor), which is an issue in both methodologies, thus affecting phylogenetic inference [42,43,44]. Nevertheless, the combined application of both genotyping techniques and the use of an 8-*loci* VNTR panel decreases the inaccuracy of genetic-relatedness inference [42,43]. The type of inconsistent membership of clades we observe between the SNP-based tree and the dendrogram may be due to homoplasy in VNTR *loci,* suggesting there is a closer evolutionary relationship between isolates than there is in reality. For six strains, the spoligotyping profile obtained by the reverse-hybridization method in the wet lab is not the same as the profile determined in silico through the vSNP pipeline, a finding that has also been reported by others (Supplementary Table S1) [45,46]. This mismatch might contribute to discrepancies between the phylogenetic tree topology based on SNP data and the dendrogram generated with classical genotyping methods.

The SNP-based phylogeny suggests geographic clustering to some extent, with clade C apparently confined to Castelo Branco and clade E being only present in Castelo Branco and Portalegre. These results are in agreement with the general knowledge of *M. bovis* clustering behavior [47,48] and with previous work performed on this *M. bovis* population from the same geographical settings that was based on the molecular characterization of isolates by classical genotyping methods [18]. This previous study suggested the existence of five *M. bovis* ancestral populations with geographic specificities. However, the Mantel testing examining the correlation between spatial and genetic distance in Castelo Branco and Portalegre were insignificant for clustering. The geographic distribution of SNP clades here registered is surely sensitive to the sampling strategy and sample size, so our results are likely due to the fact that several more distantly related lineages are present in the same region and the low number of isolates might be over exaggerating how common those other lineages are, thus affecting any signal of spatial autocorrelation. Therefore, a larger dataset, with more samples from each area, could help confirm the geographic specificities of each SNP clade and improve clustering metrics. Furthermore, a closer analysis into the temporal evolution of the SNP network evidenced strong local dynamics within *M. bovis* strains from the same geographic region, highlighting higher mean values of SNPs in common compared with the links established between *M. bovis* strains from different geographic regions. As expected, the maximum values of common shared SNPs occurred between *M. bovis* from the bordering regions of Castelo Branco and Portalegre.

Based on this and previous reports, there seems to be different roles exerted by different species depending on the region investigated. Previous molecular analyses combining spoligotyping and MIRU-VNTR, as well as WGS from this work, suggest more links with wildlife in Castelo Branco, whereas in Portalegre more cattle involvement is apparent. The sports hunting industry has fueled high densities of wild boar and red deer in the Castelo Branco district, an agroforestry region, which thus may be more likely to experience transmission from wildlife, whereas in the rural settings of Portalegre, transmission involving extensively managed cattle may be more frequent. Other studies have also shown that the role of wildlife changes by location [23,49,50,51,52,53,54]. These hypotheses are to be further demonstrated in future studies.

The TB eradication program implemented in Portugal is based on active and passive surveillance, but exclusively applied to the cattle population. In contrast, surveillance in wildlife is exclusively passive and dependent upon irregular sanitary evaluation of hunter-harvested animals, meaning that *M. bovis* infection and underlying transmission might be established for long periods of time before sample collection and molecular characterization of isolates occurs, therefore limiting transmission reconstruction inferences.

Beja is the most homogeneous region, presenting a lower mean value of SNP distance (244 SNPs); however, it must be reminded that only six isolates were analyzed from this region, while Castelo Branco and Portalegre, more represented, presented more diverse *M. bovis* populations, with a higher mean SNP distance between strains (*n* = 376 for Castelo Branco and *n* = 405 for Portalegre). Most probably, these large genetic distances among some isolates reflect introductions of new, more distantly related lineages into the same geographic regions. The introduction and dispersion of new lineages might be connected to cattle movement, wildlife translocation for commercial hunting purposes, and also to different natural movement dynamics of wildlife hosts. In some regions of Portugal, including these TB hotspots, wild boar and deer are philopatric and previous work has related wild boar to long range dissemination of infection [9,10]. Additionally, recent work based on *M. caprae* in Portugal evidenced the importance of the analysis of intra- and inter-herd dynamics to understand pathogen transmission and persistence [55]. Therefore, a detailed attention to the heterogeneity of host movement dynamics across the landscape could provide clues for geographic distribution and dispersion of clades in Portugal. WGS will greatly improve the quality of surveillance and outbreak investigations in Portugal. Future work conducted with a larger dataset and including data concerning contact patterns and cattle movement would be helpful to clarify epidemiological links.

A previous molecular approach based on two types of genomic region-direct repeats and tandem repeats [18] suggested that the *M. bovis* population from Beja shares a common ancestor with all other isolates. Again, a larger dataset would be necessary to further investigate if Beja populations are more ancestral. When considering host species, zero SNP differences involving the same wildlife species or two wildlife host species suggest the occurrence of intra- and inter-specific transmission events, supporting the importance of both wildlife species in *M. bovis* dissemination. Consequently, focus on these two wild hosts and related parameters, such as density, behavior, interaction with livestock and movement, should be considered when designing new interventions aiming to improve control of animal TB.

The SNP diversity observed within this dataset supports the natural circulation of *M. bovis* for a long period of time, as well as multiple introduction events of the pathogen in hotspots of disease in this Iberian multi-host system. In order to further investigate this emerging theme in animal TB research, WGS of larger local *M. bovis* datasets is granted in the future to clarify regional asymmetries and heterogeneous dynamics at play exerted by different species and enable finer resolution of transmission cascades. While wildlife is important, the movement of multiple lineages around Portugal might also be linked to cattle movement—so that needs future investigation too.

## 5. Conclusions

There is clearly a need to characterize *M. bovis* lineages using clade-defining SNPs. Although limited to a modest dataset, this pilot work confirms and reinforces the value of WGS application to the study of *M. bovis* transmission and persistence at the livestock-wildlife interface. The combination of WGS data and epidemiological information provided insights into the *M. bovis* demographic history in a multi-host system, enabling the analyses of recent transmission events in a few situations.

The knowledge of disease status and routes of transmission in wildlife are crucial in designing and implementing effective control measures. The findings reported in this work contribute to supporting the idea that eradication actions in the wildlife population are increasingly necessary as they can play an important role in disease transmission, sharing closely related bacterial isolates, as we demonstrate here. Altogether, we provide quantitative evidence that future control measures in livestock production systems must not ignore wildlife-related parameters, such as abundance, behavior and interaction with livestock, with the possibility for a differential approach regarding red deer and wild boar. Furthermore, our fine-scale molecular analyses suggest that Castelo Branco and Portalegre are to be considered priority areas of research and intervention and that adjacent livestock populations are to be tested more frequently.

Future WGS studies with a larger dataset from Castelo Branco and Portalegre, and from a broader time period, could help resolve global and local transmission networks, potentially enabling evolutionary analyses with estimation of evolutionary parameters, such as substitution rates, the probability of host species transition, time to the most recent common ancestor and timescales for clade divergence. Whole genome sequencing of *M. bovis* from across the world is utterly needed, not only to enlighten epidemiological scenarios, but also to build experience and tools to deal with the characteristic lack of temporal signal when slow-evolving, latent microorganisms are involved. Most algorithms and simulation tools were originally developed for the evolutionary analyses of fast-evolving microorganisms (e.g., RNA viruses) and often are inadequate to deal with molecular data from highly clonal, monomorphic organisms. Future studies with a larger dataset could give further support to the use of SNP monomorphic sites as phylogenetic markers in settings where WGS may not be easily implemented, and also contribute to understanding pathogen adaptation processes to hosts.

## Figures and Tables

**Table 1:** Number of *M. bovis* strains within each SNP clade (A to E) and identification of clade-defining and clade-monomorphic SNP sites.

SNP Clade	Total SNP Sites	Clade-Defining SNP Sites ^(a)^	Clade-Monomorphic SNP Sites ^(b)^
A (*n* = 14)	622	108	-
B (*n* = 11)	611	133	-
C (*n* = 3)	320	184	49
D (*n* = 6)	372	217	82
E (*n* = 10)	431	360	352
A to D (*n* = 34)	1419	1411	106

^(a)^ Polymorphic positions present only in the clade members. ^(b)^ Polymorphic positions present only in the clade members and common to all members.

## Data Availability

Data sharing will be granted by the corresponding author upon reasonable request. The raw data are deposited in a public domain server at the NCBI SRA database, under BioProject accession number PRJNA682618.

## Supplementary Materials

The following are available online at https://www.mdpi.com/article/10.3390/microorganisms9081585/s1, Figure S1: Minimum spanning tree (MST) illustrating genetic relationships among *M. bovis* (*n* = 487) based on spoligotyping and 8-*loci* MIRU-VNTR data, using single locus variant analysis. Figure S2: Timeline of animal tuberculosis in Portugal. Figure S3: Temporal distribution of *M. bovis* identified by SNP clade per year. Table S1: Characteristics of *Mycobacterium bovis* selected for WGS. Table S2: *Mycobacterium bovis* sequencing statistics details.
