# Supplementary material for: Whole Genome Sequencing Refines Knowledge on the Population Structure of Mycobacterium bovis from a Multi-Host Tuberculosis System
Source: Microorganisms. 2021 Jul 26;9(8):1585. doi: 10.3390/microorganisms9081585 (PMC8401292; doi:10.3390/microorganisms9081585)
Supplement: Supplementary file 1 [file microorganisms-09-01585-s001.zip › microorganisms-1316601-supplementary.pdf]

Whole Genome Sequencing refines knowledge on the population structure of *Mycobacterium bovis* from a Multi-Host Tuberculosis System

Ana C Reis [1,2], Liliana C.M. Salvador [3,4,5], Suelee Robbe-Austerman [6], Rogério Tenreiro [2], Ana Botelho [7], Teresa Albuquerque[7], Mónica V. Cunha[1,2*]

[1] Centre for Ecology, Evolution and Environmental Changes (cE3c), Faculdade de Ciências da Universidade de Lisboa, Lisboa, Portugal; ana.reis714@gmail.com; mscunha@fc.ul.pt
[2] Biosystems & Integrative Sciences Institute (BioISI), Faculdade de Ciências da Universidade de Lisboa, Lisboa, Portugal; rptenreiro@fc.ul.pt
[3] Department of Infectious Diseases, College of Veterinary Medicine, University of Georgia, Athens, Georgia, USA; salvador@uga.edu
[4] Institute of Bioinformatics, University of Georgia, Athens, Georgia, USA
[5] Center for the Ecology of Infectious Diseases, University of Georgia, Athens, Georgia, USA
[6] USDA/APHIS National Veterinary Services Laboratories, USA; suelee.robbe-austerman@usda.gov
[7] INIAV, IP - National Institute for Agrarian and Veterinary Research, Portugal; ana.botelho@iniav.pt; teresa.albuquerque@iniav.pt
* Correspondence: mscunha@fc.ul.pt; Tel.: (+351) 217 500 000, ext. 22461

Supplementary Information

Supplementary Table S1. Characteristics of *Mycobacterium bovis* selected for WGS.

M. bovis ID	Sampling date	Host species	Geographic region	Spoligotype[a]	*In silico* spoligotype	MIRU-VNTR profile								Ancestral Population[b]
						VNTR 3232	ETR-A	ETR-B	ETR-C	QUB 11a	QUB 11b	MIRU 26	MIRU 4	
Mb0220	2003	Cattle	Portalegre	SB1174	SB1174	6	4	5	4	11	4	3	5	AM3
Mb0261	2006	Red deer	Castelo Branco	SB0120	SB0265	4	5	4	3	11	2	3	5	AM5
Mb0601	2007	Cattle	Beja	SB0295	SB0295	4	6	3	4	8	2	3	5	AM2
Mb0754	2008	Cattle	Portalegre	SB0121	SB0121	4	7	4	5	11	2	3	5	AM1
Mb0769	2008	Cattle	Beja	SB0119	SB0119	4	6	4	4	11	2	2	5	AM1
Mb0783	2008	Wild boar	Castelo Branco	SB0121	SB0122	4	6	3	4	11	3	3	5	AM2
Mb0865	2008	Cattle	Castelo Branco	SB1090	SB1090	4	6	4	2	>12	2	3	2	AM4
Mb0891	2009	Red deer	Castelo Branco	SB1264	SB1264	4	6	4	2	11	2	3	5	AM5
Mb0893	2008	Wild boar	Castelo Branco	SB0119	SB0121	4	6	4	4	11	3	3	5	AM2
Mb1317	2010	Cattle	Beja	SB0265	SB0265	4	1	4	3	11	2	3	5	AM5
Mb1339	2010	Cattle	Castelo Branco	SB0122	SB0122	4	6	3	4	11	3	3	5	AM2
Mb1458	2010	Wild boar	Castelo Branco	SB1232	SB1174	4	5	5	4	11	4	3	5	AM3
Mb1480	2010	Cattle	Portalegre	SB1174	SB1174	4	4	5	4	11	4	3	5	AM3
Mb1654	2011	Cattle	Portalegre	SB0121	SB0121	4	5	4	4	11	2	3	5	AM1
Mb1670	2011	Red deer	Castelo Branco	SB1174	SB1174	4	5	5	4	11	4	3	5	AM3
Mb1711	2011	Red deer	Castelo Branco	SB1264	SB1264	4	5	4	4	11	2	2	5	AM1
Mb1712	2011	Red deer	Castelo Branco	SB1195	SB1195	4	6	4	2	11	2	3	5	AM5

Mb1714	2011	Cattle	Castelo Branco	SB0265	SB0265	4	5	4	2	11	2	3	5	AM5
Mb1744	2012	Wild boar	Castelo Branco	SB1264	SB1264	4	5	4	4	11	2	3	4	AM1
Mb1746	2012	Red deer	Castelo Branco	SB0121	SB0121	4	6	4	4	11	2	3	5	AM5
Mb1758	2012	Cattle	Portalegre	SB1264	SB1264	4	5	4	4	11	2	3	5	AM1
Mb1769	2012	Wild boar	Castelo Branco	SB1195	SB1195	4	6	4	2	11	2	3	5	AM5
Mb1785	2012	Red deer	Beja	SB1190	SB1190	4	6	4	4	11	2	3	5	AM5
Mb1789	2012	Cattle	Portalegre	SB1264	SB1264	4	5	4	4	11	2	2	5	AM1
Mb1841	2012	Cattle	Portalegre	SB0121	SB0121		5	4	4	11	2	3	5	AM1
Mb1870	2012	Wild boar	Portalegre	SB1264	SB1264	4	5	4	4	11	2	3	5	AM1
Mb1915	2013	Red deer	Castelo Branco	SB0265	SB0265	4	5	4	2	11	2	3	5	AM5
Mb1948	2013	Red deer	Castelo Branco	SB1174	SB1174	4	4	5	4	11	4	3	5	AM3
Mb1960	2013	Red deer	Castelo Branco	SB1264	SB1264	4	5	4	4	11	2	3	5	AM5
Mb2026	2013	Cattle	Portalegre	SB1095	SB1190	-	6	4	4	11	2	3	5	AM5
Mb2043	2013	Red deer	Portalegre	SB1264	SB1264	4	7	4	4	11	2	2	5	AM1
Mb2067	2013	Wild boar	Beja	SB1190	SB1190	4	6	4	4	11	2	3	5	AM5
Mb2206	2014	Cattle	Castelo Branco	SB1190	SB1190	5	6	4	4	11	2	3	5	AM1
Mb2235	2014	Red deer	Portalegre	SB1232	SB1174	-	4	5	4	11	4	3	5	AM3
Mb2267	2014	Cattle	Portalegre	SB1174	SB1174	4	4	5	4	11	4	3	5	AM3
Mb2277	2014	Red deer	Portalegre	SB1174	SB1174	4	4	5	4	11	4	3	5	AM3
Mb2300	2014	Wild boar	Portalegre	SB0121	SB0121	4	5	4	4	11	2	3	5	AM1

Mb2310	2015	Red deer	Portalegre	SB0122	SB0122	4	5	3	4	11	3	3	5	AM2
Mb2313	2015	Wild boar	Castelo Branco	SB0265	SB0265	4	5	4	2	11	2	3	5	AM5
Mb2325	2015	Red deer	Beja	SB0265	SB0265	4	1	4	2	11	2	3	5	AM5
Mb2328	2015	Red deer	Castelo Branco	SB0122	SB0122	4	6	3	4	11	3	3	5	AM2
Mb2347	2015	Wild boar	Portalegre	SB1174	SB1174	4	4	5	4	11	4	3	5	AM3
Mb2395	2015	Wild boar	Castelo Branco	SB0121	SB0121	4	6	4	4	11	2	3	5	AM5
Mb2397	2015	Wild boar	Castelo Branco	SB0121	SB0121	5	6	4	4	11	2	3	5	AM1

(a) Spoligotyping profile obtained by reverse hybridization method

(b) Classification in ancestral population (AM1 to AM5) as described in Reis et al., 2020.

Supplementary Table S2. *Mycobacterium bovis* sequencing statistics details.

M. bovis ID	Sampling date	Host species[(a)]	Geographic region[(b)]	R1size	R2size	Allbam mapped reads[(c)]	Genome coverage[(d)]	Average coverage	Average read length	Unmapped reads	Unmapped assembled contigs
Mb0220	2003	C	PG	217.8MB	230.4MB	2394434	99.69	126.7	238.1	25077	2
Mb0261	2006	RD	CB	448.7MB	466.7MB	13725892	99.92	368.8	151.0	2146326	13966
Mb0601	2007	C	BJ	171.7MB	204.8MB	191835	99.73	98.7	234.2	22676	32
Mb0754	2008	C	PG	159.0MB	171.9MB	1359222	99.64	72.5	240.8	9193	4680
Mb0769	2008	C	BJ	185.4MB	215.7MB	2047981	99.68	108.1	240.2	19903	3
Mb0783	2008	WB	CB	191.2MB	221.1MB	2012194	99.66	105.5	237.8	22613	384
Mb0865	2008	C	CB	151.7MB	162.1MB	1410917	98.79	73.7	242.2	11861	366
Mb0891	2009	RD	CB	404.8MB	419.0MB	13142182	99.91	345.4	151.0	1898427	74
Mb0893	2008	WB	CB	169.3MB	180.7MB	188456	99.66	100.6	241.1	15639	4
Mb1317	2010	C	BJ	157.2MB	161.8MB	1707221	99.59	91.3	241.5	13657	1
Mb1339	2010	C	CB	152.4MB	172.9MB	1640461	99.63	87.2	240.5	13024	1
Mb1458	2010	WB	CB	186.3MB	216.5MB	192992	99.67	103.1	242.2	19592	2
Mb1480	2010	C	PG	192.4MB	205.2MB	205313	99.64	109.7	240.2	19865	2273
Mb1654	2011	C	PG	117.7MB	130.9MB	615194	99.34	33.3	236.7	3968	60498
Mb1670	2011	RD	CB	189.5MB	206.5MB	2074834	99.64	110.1	239.7	20213	121
Mb1711	2011	RD	CB	161.6MB	168.0MB	175829	99.64	93.2	238.1	12588	88
Mb1712	2011	RD	CB	121.4MB	129.6MB	1085013	99.05	55.6	241.4	9286	2030
Mb1714	2011	C	CB	101.8MB	110.3MB	788996	99.37	40.5	241.3	7143	11013
Mb1744	2012	WB	CB	172.5MB	188.0MB	1796448	99.69	92.5	235.1	14316	3
Mb1746	2012	RD	CB	165.0MB	180.6MB	1728552	99.7	88.1	233	12827	1
Mb1758	2012	C	PG	163.2MB	175.3MB	1732898	99.52	93.4	241.8	14928	1412
Mb1769	2012	WB	CB	135.4MB	143.5MB	1374917	99.15	72	238.6	8851	1
Mb1785	2012	RD	BJ	115.5MB	120.4MB	940707	99.56	49.5	240	5925	9156
Mb1789	2012	C	PG	125.4MB	133.9MB	970309	99.49	50.8	234.3	6378	811

Mb1841	2012	C	PG	168.9MB	191.2MB	176723	99.67	91.2	237.4	15809	1
Mb1870	2012	WB	PG	130.8MB	133.8MB	1395146	99.64	67.7	223	12333	30
Mb1915	2013	RD	CB	137.6MB	155.4MB	1423501	99.65	74	239	11027	5
Mb1948	2013	RD	CB	153.1MB	157.7MB	1523134	99.66	79.4	237.3	10609	2
Mb1960	2013	RD	CB	158.6MB	177.5MB	1712114	99.55	91.3	239.6	13976	1584
Mb2026	2013	C	PG	134.4MB	144.7MB	1236634	99.61	64.2	243	12192	557
Mb2043	2013	RD	PG	113.7MB	121.1MB	966319	99.41	50.3	241.9	7475	548
Mb2067	2013	WB	BJ	178.9MB	193.9MB	2004187	99.71	105.1	236	19496	283
Mb2206	2014	C	CB	162.3MB	184.7MB	1751017	99.72	92.7	238.1	14763	2505
Mb2235	2014	RD	PG	174.0MB	188.8MB	1891547	99.65	100.4	238.9	16555	346
Mb2267	2014	C	PG	168.4MB	185.0MB	890928	99.52	46.9	234.6	6772	4287
Mb2277	2014	RD	PG	68.6MB	73.6MB	586402	99.36	29.8	241.5	4986	258
Mb2300	2014	WB	PG	256.2MB	268.0MB	2833579	99.75	149.2	237.7	36471	38
Mb2310	2015	RD	PG	132.8MB	146.4MB	1290912	99.63	65.8	234.6	8582	1136
Mb2313	2015	WB	CB	164.8MB	186.8MB	1671813	99.65	85.3	238.3	17263	1
Mb2325	2015	RD	BJ	162.0MB	185.5MB	1687836	99.67	86.7	237.6	15786	2
Mb2328	2015	RD	CB	129.9MB	150.0MB	1220275	99.48	65.4	240	9374	15533
Mb2347	2015	WB	PG	108.9MB	124.5MB	1135051	99.56	60.4	239	8944	465
Mb2395	2015	WB	CB	216.4MB	239.9MB	2365469	99.74	124.4	237.3	27435	3
Mb2397	2015	WB	CB	203.2MB	215.0MB	2171022	99.58	116.4	240.9	20636	1

(a) C - Cattle; RD - Red deer; WB - Wild boar

(b) Beja - Beja; CB- Castelo Branco; PG - Portalegre

(c) Number of successfully assembled, trimmed paired-end Illumina reads.

(d) Relative to *M. bovis* reference genome AF2122/97 (NCBI accession number LT708304.1).

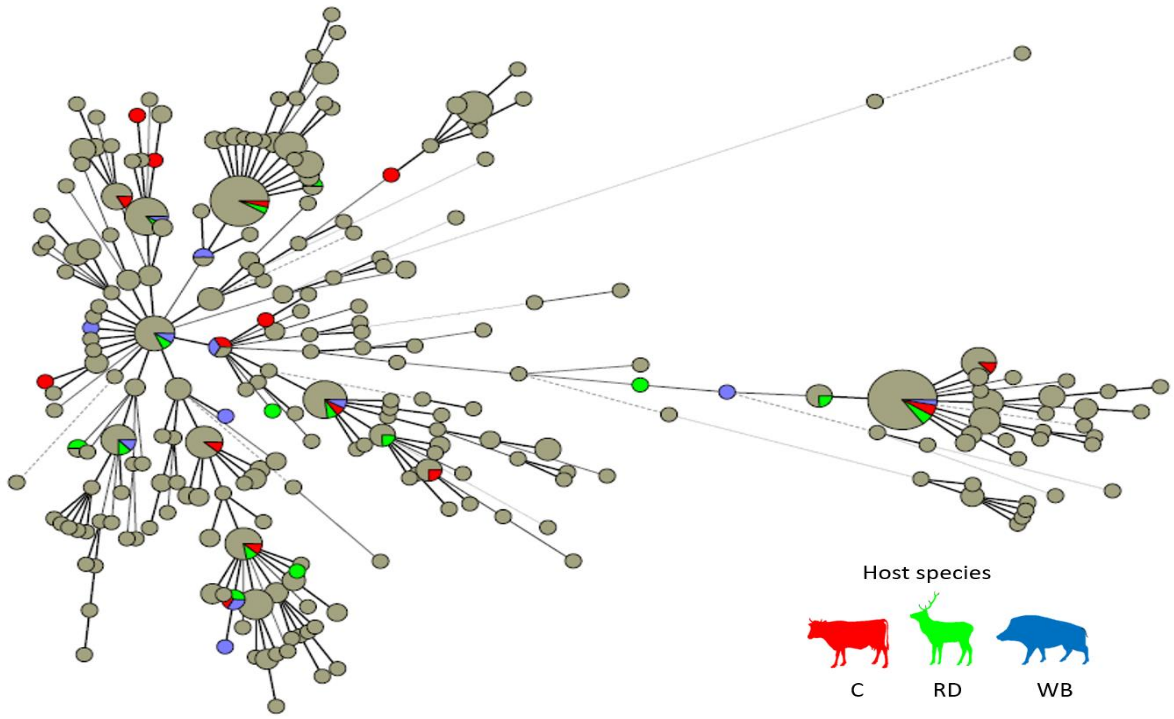

Supplementary Figure S1. Minimum spanning tree (MST) illustrating genetic relationships among *M. bovis* (*n*=487) based on spoligotyping and 8-*loci* MIRU-VNTR data, using single *locus* variant analysis. Circle size is proportional to the number of isolates within each node; and colours represent hosts species (cattle - red, red deer - green and wild boar - blue). The complexity of the lines denotes the number of differences in the spoligo-MIRU type profile between two nodes: solid lines (1, 2 or 3 differences), grey dashed lines (4 differences) and grey dotted lines (5 or more differences). Only *M. bovis* selected for WGS are coloured.

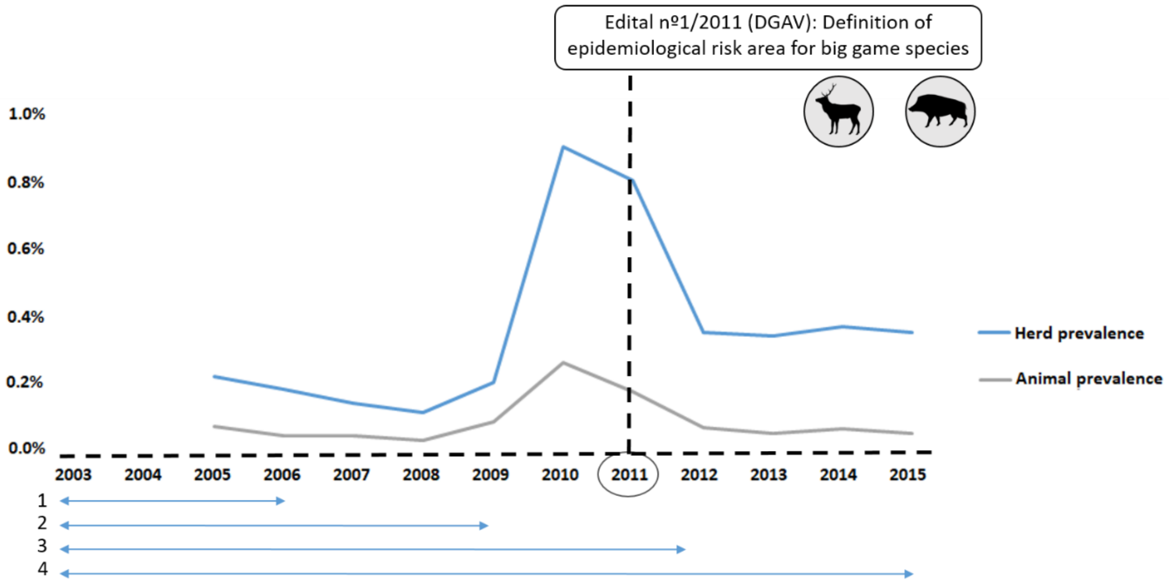

Supplementary Figure S2. Timeline of animal tuberculosis in Portugal. The curves represent the evolution of animal TB epidemiological indicators, for cattle population, in mainland Portugal (2003-2017) [Adapted from Relatório Técnico de Sanidade Animal, DGAV (2015)]. The year 2011 marks the definition of epidemiological risk area for big game species. The cumulative time periods considered in this work are evidenced under the timeline. Periods 1 and 2 were pooled to achieve adequate sample sizes.

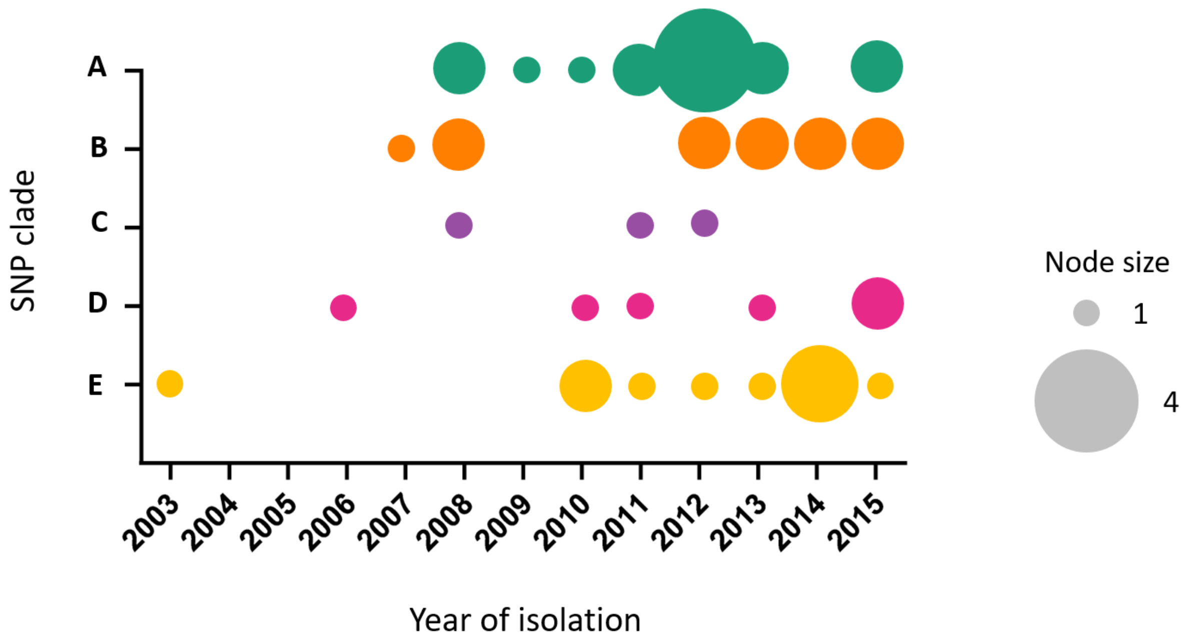

Supplementary Figure S3. Temporal distribution of *M. bovis* identified by SNP clade per year. Node size is proportional to the number of *M. bovis* strains within each node.
